# Academy of Stomatology

**Published:** 1903-04

**Authors:** 

**Affiliations:** Academy of Stomatology


### ACADEMY OF STOMATOLOGY.

A REGULAR monthly meeting of the Academy of Stomatology of Philadelphia was held at its rooms, 1731 Chestnut Street, on the evening of Tuesday, October 28, 1902, the President, Dr. R. Hamill D. Swing, in the chair.

A lecture was given by Dr. L. C. Custer, of Dayton, Ohio, on "The Practical Use of the X-Ray in Dentistry." Dr. Custer illustrated his lecture by the use of charts, skiagraphs, and by a practical demonstration of his apparatus and its use on the actual patient.

(For Dr. Custer's paper, see page 247.)

A paper was read by Dr. Water H. Neall, of Philadelphia, entitled "Notes on Mummification of the Dental Pulp."

(For Dr. Neall's paper, see page 251.)

DISCUSSION ON DR. CUSTER'S PAPER.

*Dr. Charles L. Leonard.*—I have been much interested in the doctor's demonstration and have learned something of value in the application of the X-ray in dental work. I have used the X-ray in the line of stomatology. In cases of ankylosis of the jaw, in which the patients were unable to open the mouth but to a very slight extent, I have by skiagraphy demonstrated the ankylosis on both sides. The patients were successfully operated upon and motion restored in each case. In another case in the line of the dental surgeon, and in which I was called in to assist, the patient fell through an elevator shaft, causing a broken jaw. Union was defective, and an examination by the X-ray showed the root of the tooth protruding in the line of fracture in the jaw. Removal of the tooth and wiring together of the molars facilitated complete restoration of motion between the fragments, and the bone united satisfactorily. In a number of cases I have examined patients for supposed non-erupted teeth. In one case there was shown the presence of necrosis of the jaw-bone. Operation demonstrated the presence of abscess and relieved the patient from the condition.

The therapeutic use of the X-ray is a new field which is widening and developing every day. We hardly know to-day what application of it will be made to-morrow. It has come within my knowledge to-day to know of an operation which Dr. Keen told me he had done at the Jefferson College Hospital. He covered the half of a sarcoma of the breast with lead, which intercepts the X-ray, and the other half he gave ten or twelve treatments with the X-ray. After removal of the tumor microscopical examination showed a complete degenerative process, showing that we possess in the X-ray a therapeutic agent which is capable not only of restricting the development of malignant disease, but of actually destroying it, and that we have in it an agent more potent therapeutically than any other agent known for the control of malignant disease. Before coming here this evening I was treating a patient with carcinoma of the floor of the mouth. It shows above the alveolar border almost to the level of the upper teeth. When the patient came under treatment he had great pain. The swelling was so great that he was unable to eat. He had also, and has still, a stoppage of the salivary ducts and swelling of those salivary glands on either side beneath the maxilla.

The pain has disappeared entirely and only recurs slightly on the day before he comes for treatment. He has only been given six treatments. The growth has not increased, nor has there been any marked decrease in its size. There is, however, relief from pain. I have seen the same thing occur in sarcoma of the breast to such an extent that morphia has been entirely unnecessary, and the patient has increased in weight and gained in strength without the disorders incident to the use of morphia.

The therapeutic side of the use of this agent is therefore one of the utmost value. It is only in certain cases that it would come within the range of the stomatologist. I have had patients ask for relief of neuralgias of the inferior dental nerve or of the superior nerve. That is a condition which the treatment does not seem to affect, but it has not been tried, I think, with sufficient diligence.

In malignant disease we have in early and radical operation a treatment which in a large percentage of cases, if it does not give freedom from recurrence, at least gives the patient the longest lease of life. Until we can prove that the X-ray can give as good results, I think it is our duty to give the patient the advantages of operation and follow that by the X-ray treatment. That is, the macroscopic tumor should be removed if possible and the microscopic remnants which the knife does not touch can be treated by the X-ray and the patient thus given the double chance from the two remedies which we know to be most efficient.

The doctor has shown us an apparatus which seems to me as perfect as one can desire. It lacks, however, a certain variability, a certain power of alteration which is requisite in the more difficult forms of diagnosis and in therapeutic work. In certain conditions in the dental work we are dealing with opacities which are very great, and with shadows one of which is greater than the other. The bone is less dense than the tooth, and in order to penetrate the tooth you have to use a very high vacuum light which has great power in penetration. The doctor has demonstrated how perfectly that is done. He has shown what light is sufficient and proper for that purpose. It has the added advantage of shortness of exposure and the efficiency of that exposure without danger of X-ray burn. I was speaking to the doctor before the meeting of a case in New York to which I was called as an expert by the dentist there. The dentist was sued for an

X-ray burn, the result of an examination for a suspected fragment of molar. I think the burn was due to the condition of the patient at the time. We know that under unknown conditions the X-ray burn is produced. The time and distance of the tube were such as I would have used if they had not even run into more dangerous conditions. We attribute such a burn to the idiosyncrasy of the individual. I think, however, it is not so much that as it is owing to the state of the individual at the time the examination is made. I believe we produce with the X-ray stimulation of the vitality of normal tissue and over-stimulation of pathological tissue. We know also that pathological tissue is liable to necrosis and death from over-stimulation by a stimulus not too great for normal tissue. So that we have here the helping of Nature in the stimulation of normal tissue and in the over-stimulation of pathological tissue. You see how a patient's depressed condition would account for a burn under conditions that would not produce burn in the normal state of that individual. It is that which we have to study very carefully in therapeutic work. You have to test the condition of the patient and test the effect of the X-ray before beginning treatment. When used in that way the X-ray seems to have an effect which nothing else has. We look forward to its future, of which none can determine actually its value. Those who are studying in it can see farther than those who are simply onlookers. Yet, those who are in it do not know where the value of it will end.

*Dr. Kassabian.*—Dr. Custer's able demonstration of the technique of the X-ray in dentistry leaves little for me to add to the subject except perhaps a few remarks regarding the importance of X-ray diagnosis in dentistry, in the hope that more enthusiasm may be aroused in the dental profession. My own experience has been practically limited to surgical cases. Out of six thousand skiagraphs, only one hundred were dental cases. There is not in Philadelphia a single dentist who applies the X-ray systematically as do Drs. Custer, Price, and Kells in other cities. I firmly believe that X-ray diagnosis in dental surgery must be made by a dentist who understands the requirements thoroughly, not only from the stand-point of the X-ray technique, but also from the stomatological stand-point. It is a simple and easy operation to take a dental skiagraph, as Dr. Custer has demonstrated, and there is no more danger to the patient in submission to its proper use than in riding in a railroad car. In all my cases I never burned a patient, but the repeated exposure of my own hands to the effect of the ray has produced in them an X-ray dermatitis which does not yield readily to ordinary treatment. Repeated and long exposures to the ray may cause severe burn. My own exposures are very short.

In closing I would pay a word of tribute to Professor Röntgen, who is ranked with other great discoverers such as Pasteur, Lister, etc.

(The doctor exhibited some interesting skiagraphs.)

*Dr. M. H. Cryer.*—I have not much to say except that I have been unfortunate so far in having some of our best X-ray men take skiagraphs for me. To-day if I had a case of impacted tooth I would depend upon a good sharp excavator rather than upon the X-ray. I am sorry that Dr. Custer does not live here. I would have had his assistance in a case of caries of the lower jaw extending from the symphysis back to the condyloid process. I have taken the patient to a rather celebrated man in this city, but have secured no results. I cannot tell exactly how far the necrosis extends, but I am waiting, hoping that the periosteum is building new bone under the dead bone. The mouth is not in a healthy condition, and it is a question how long I shall let this dead bone remain. If I remove it before new bone is formed I shall have a deformity. If the X-ray would show me that new bone is being formed under the dead bone I would be inclined to let that remain until there were symptoms of blood-poisoning from the bones there. I think the instrument is not one for the dentist to use without having some means to protect his hands. Many of you know that I am fond of photography, and I was inclined to take it up, but when I saw the hands of some of my friends who work in it I concluded I could not afford to have my hands in that condition.

*Dr. Joseph Head.*—This most interesting and valuable demonstration seems to me to have a most practical side to it. We are, of course, very much pleased to be able to see the impacted wisdom-tooth and to know when a tooth is or is not below a temporary molar, or whatever it may be that we wish to investigate, but it seems to me that the side so clearly brought forward by Dr. Leonard should receive our special attention. For years dentists and doctors have been looking askance at every tumor that comes into the mouth and feeling that it had better be left alone. Tumors which were benign have become malignant. It has been my experience in several instances to be compelled to remove these tumors, and I always do it with a certain amount of feeling of responsibility for the injury that might come to the patient should it prove to be malignant. In the light of our present knowledge it seems to me that instead of accepting a doubtful responsibility there is a certain amount of compulsion placed upon all dentists that whenever a tumor appears in the mouth to take it out as thoroughly as possible instead of, as in times past, having a little piece cut from the outside for examination. The tumor should be cut into sections from beginning to end, and if signs of malignancy are shown the patient should be handed over to our friends such as Dr. Leonard represents. The mouth should be kept open and the X-ray applied to that site where the tumor existed, the rest being protected by a lead cone, and in that way the patient may be preserved with almost absolute certainty from recurrence. This might be said to be capable of performance in at least ninety-nine per cent. of cases, for I think Dr. Leonard once told me that only very occasionally does it happen that these malignant growths are stimulated rather than destroyed.

*Dr. S. H. Guilford.*—That which I have in mind is not exactly in the line of discussion, but during the past summer, while in Copenhagen, I visited an institute established for investigating purposes and for the use of the electrical rays. I went through the building, which is a fine one, with a large staff of assistants and any number of patients. The X-ray is not used, but the chemical rays of the electric light. The system is such that the doctor has as many as twenty-seven patients at a time. In the large room in which treatment is conducted is a large-sized tube, and from it four branches run off, so that each additional tube reflects light upon an operating-table upon which the patients are laid. The treatment has been very successful in lupus, acne, and diseases of that kind. Experiments are being made in the treatment of tuberculosis. One case of X-ray burn that had up to that time resisted all treatment was showing good results. The work is purely experimental and is absolutely charitable. In lupus and acne some strong acid is first used, and after that treatment is entirely by the use of the light rays and the patient subjected each day to the treatment for an hour and ten minutes. The electrical rays as they come from the arc light are passed through a chemical solution of ferric potassium. All the rays not needed are taken out, and the chemical rays are the ones which do the work.

*Dr. John C. Curry.*—Is the X-ray supposed to have antiseptic properties on infected tissue, or does it simply stimulate tissue; and has the X-ray ever been used on disease germs pure and simple?

*Dr. Custer.*—I did not quite understand Dr. Leonard regarding the range of the appliance.

*Dr. Leonard.*—I simply meant that with this method of interruption you do not evolve as much with the lower vacuum as you do with the high. It is suitable for high vacuum and for frequent interruptions. In certain forms of X-ray work it is more difficult to use this form of interrupter. The apparatus is most suitable for dental work, but I think in its general application it is not quite as useful as some other forms of interruption. That, however, is perhaps simply a matter of opinion with operators.

*Dr. Custer.*—In our dental work where the conditions are nearly all the same it is not a thing which we would take up practically, but there is no question that the range of requirements is quite extensive, and for that reason the criticism of Dr. Leonard is quite in place.

In regard to the hands, I know that one beginning the work would be inclined to overdo and get a bad condition of the hands. I have a fairly good pair of hands; the only difficulty I have had is with the condition of the nails. They have not the strength of good nails. If to a beginner the danger of exposure could be shown, much difficulty would be overcome.

The X-ray, in the first days, was supposed to be a germicide to some extent. Certainly we have reason to believe it is so.

### DISCUSSION ON DR. NEALL'S PAPER.

*Dr. M. L. Schamberg.*—I might explain that the reason I have been called upon to discuss this paper is that a few years ago I conducted some clinical experiments in pulp mummification. By way of introduction I might say that I have little regard for that man who has routine methods of treating pulps or of filling teeth. It is decidedly unprofessional. It partakes more of the nature of the work of a laborer than of the work of a man with a brain to have a routine method from which he does not vary. For that reason I was much interested in pulp mummification on account of its application in a few cases that occasionally arise in the practice of almost every dentist. In such cases as third molars, when we have exposure and when the character of the root is entirely unknown to us, or where the tooth has many roots and it is almost impossible to definitely locate these pulp-canals, or, again, when time is a great object, I think one is justified in using a pulp mummifier.

*Dr. Hickman.*—This subject appeals to me because I had two experiences with the same paste which Dr. Neall used. One of the cases was that of a very nervous patient with whom you could not do anything, and I introduced what is known as Kellogg's paste. It cured the slight pain, but it reappeared. I removed the pulp with the pressure method. In another case I had the same experience, and I have never used the paste since. A friend of mine in Los Angeles used this paste quite a good deal, and thought he had found something of value. He had no trouble for a year, when he reaped the whirlwind that Dr. Neall mentioned. He said he had abscesses in all directions. I saw him to-day, and asked him what he thought to be the best mummifier. His answer was, "A broach." Since my little experience, and from what has been told me, I have taken the pulp out and used oxychloride of zinc for canal filling.

*Dr. Neall* (closing).—The method I have used is to cleanse the pulp thoroughly, press the paste on it, and then fill with a soft amalgam. Most of the cases have been in the first molar, which is inclined to decay. The third molars have offered a good opportunity for experiment. In one case of severe exposure of the pulp the treatment was used. The gentleman has been undergoing other operations since, and so far all is satisfactory.

As I said, I do not know what the harvest will be. I know what I shall do if the whirlwind strikes some of the third molars, but with the more anterior teeth the problem is a more difficult one.

Adjourned.

OTTO E. INGLIS,
*Editor Academy of Stomatology.*

